# Supplementary material for: Killer-like receptors and GPR56 progressive expression defines cytokine production of human CD4+ memory T cells
Source: Nat Commun. 2019 May 22;10:2263. doi: 10.1038/s41467-019-10018-1 (PMC6531457; doi:10.1038/s41467-019-10018-1)
Supplement: Supplementary file 1 — Supplementary Information [file 41467_2019_10018_MOESM1_ESM.pdf]

Supplementary information

"Progressive expression of killer-like receptors and GPR56 defines cytokine production of human CD4+ memory T-cells"

Truong, Schlickeiser et al.

Supplementary figure 1: TIM-3, TIGIT, LAG-3 and PD-1 surface expression in human circulating CD4$^+$ T cell subsets

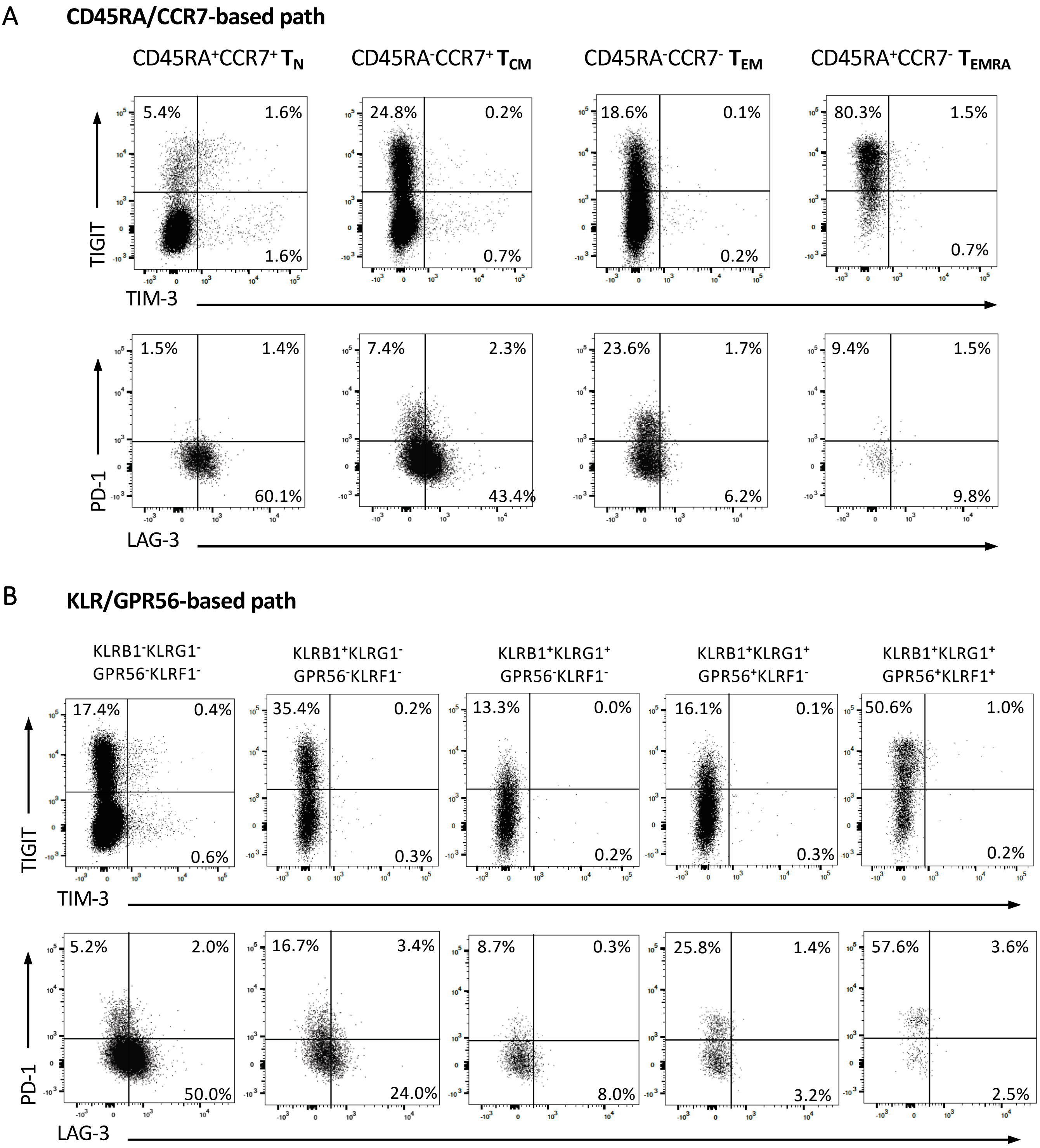

Exemplary dot plots of co-inhibitory/exhaustion receptors within (A) CD45RA/CCR7-based or (B) KLR/GPR56-based classification of human CD4$^+$ T cell subsets from blood of healthy donors.

Supplementary figure 2: Association of killer-like receptors (KLR) and GPR56 expression with IL-4 & IL-17A production upon PMA/Ionomycin stimulation

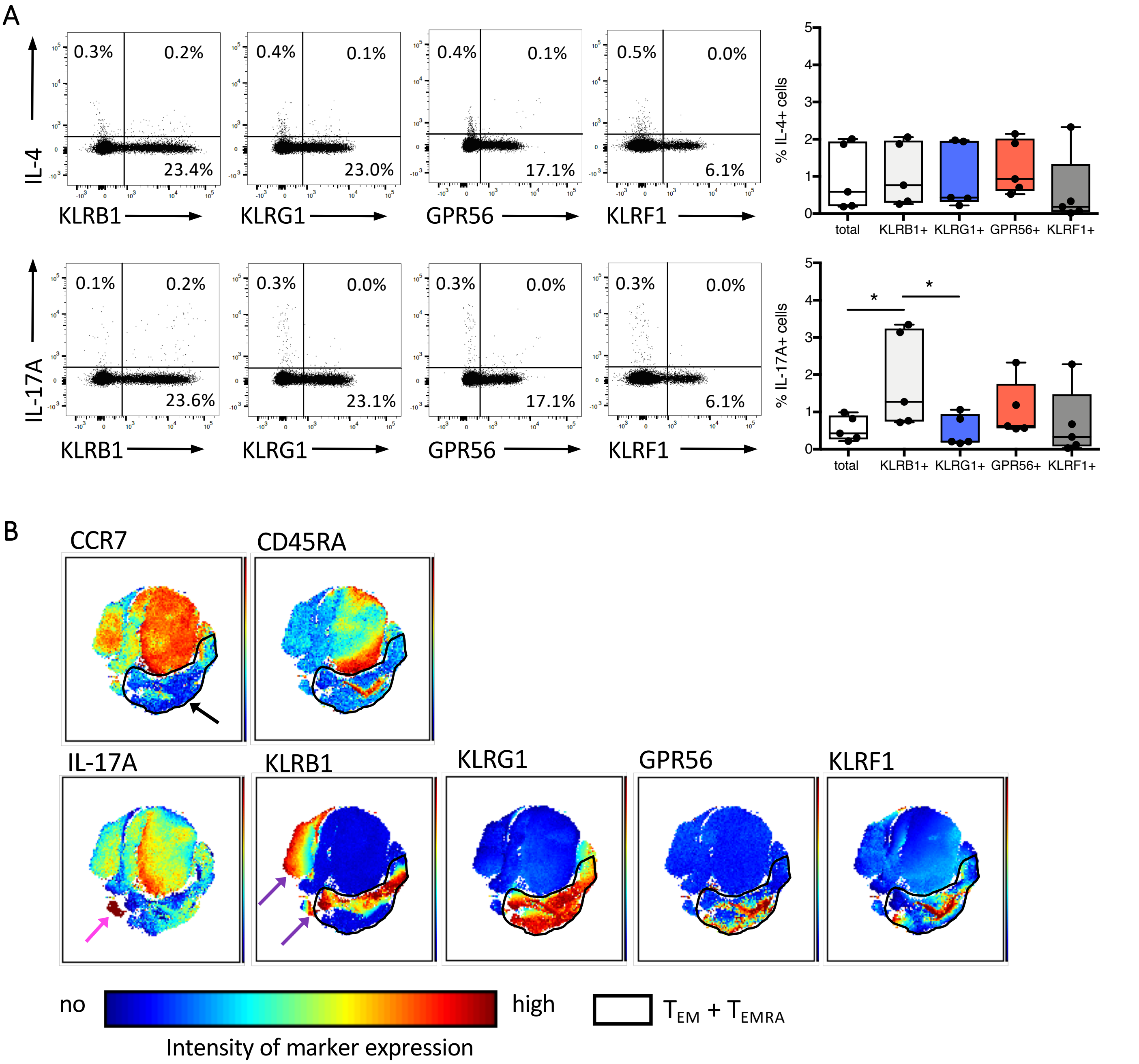

(A) Exemplary dot plots and summarising box plots revealing association of surface marker expression with IL-4 & IL-17A expression potential of $CD4^+$ T cells from peripheral blood of healthy individuals upon PMA/Ionomycin stimulation (n = 5). Whiskers extend to minimum and maximum. P values were determined by non-parametric matched-pairs Friedman test with post-hoc Dunn's multiple comparison test. *p < 0.05.
(B) Representative t-SNE plots showing surface marker and cytokine expression pattern of pre-gated $CD4^+$T cells (excluding $CD25^{high}CD127^{low}$ Treg cells) upon short-term PMA/Ionomycin stimulation. The highlighted area marks $CD4^+$ T_{EM} and T_{EMRA} cells identified by the absence of CCR7 and the expression pattern of CD45RA. The pink error identifies high IL-17A producers residing outside of the TEM + TEMRA area but showing expression of KLRB1 only.

Supplementary figure 3: Comparative analysis of IFN-g & TNF co-production in KLR / GPR56 expressing subsets upon stimulation with PMA / Ionomycin, staphylococcus enterotoxin B (SEB), tetanus toxoid (TT) or cytomegalovirus peptide (CMV)

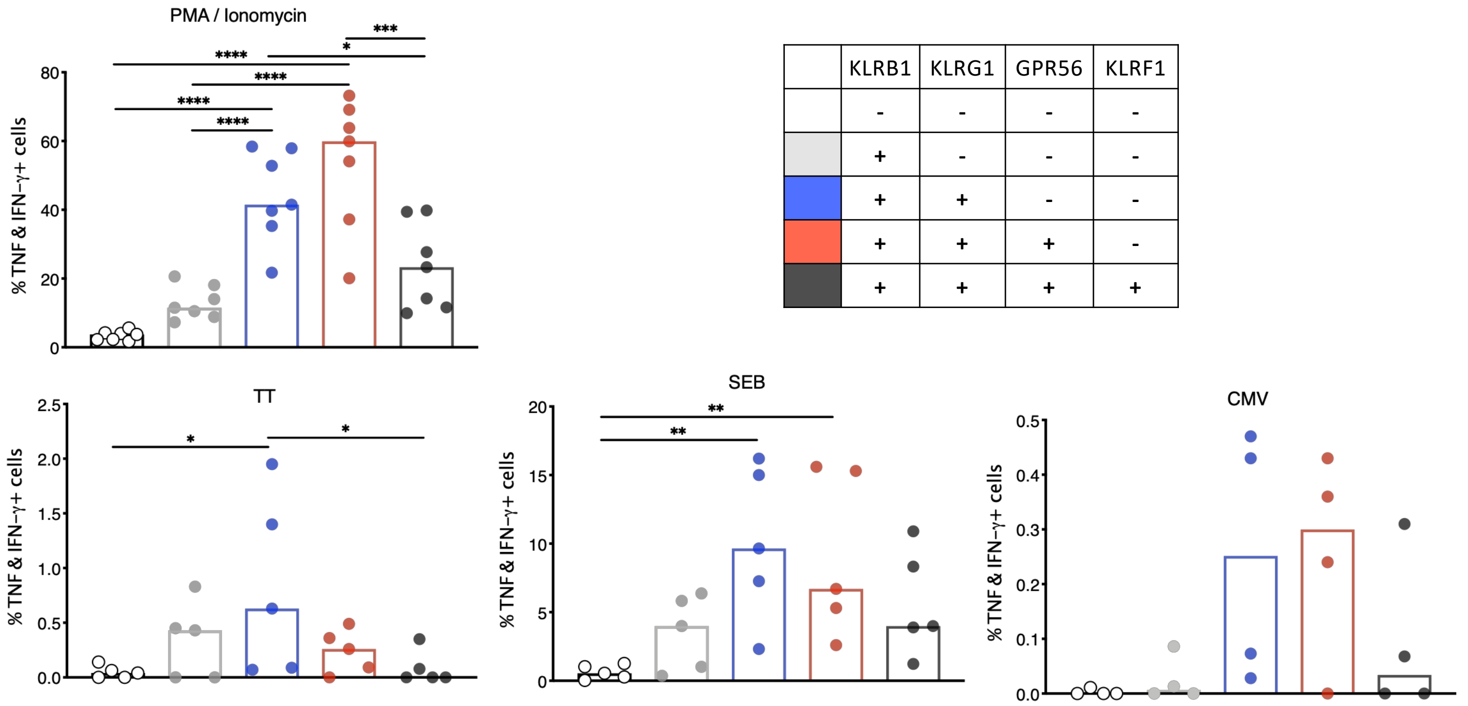

	KLRB1	KLRG1	GPR56	KLRF1
	-	-	-	-
	+	-	-	-
	+	+	-	-
	+	+	+	-
	+	+	+	+

Interleaved scatter plots with medians revealing association of surface marker expression with cytokine expression of CD4+ T cells from peripheral blood of healthy individuals upon stimulation with PMA / Ionomycin (n = 5) for six hours, SEB (n = 5), TT (n = 5) and CMV peptide (IE+pp65, n = 4) for 24 hours. *p<0.05, ***p<0.001, ****p<0.0001 (non-parametric matched-pairs Friedman test with post-hoc Dunn's correction for multiple comparison)

Supplementary figure 4: Decreased proportions of *KLRF1* transcribing T_{EM} cells in liver of patients with inflammatory biliary and hepatic diseases

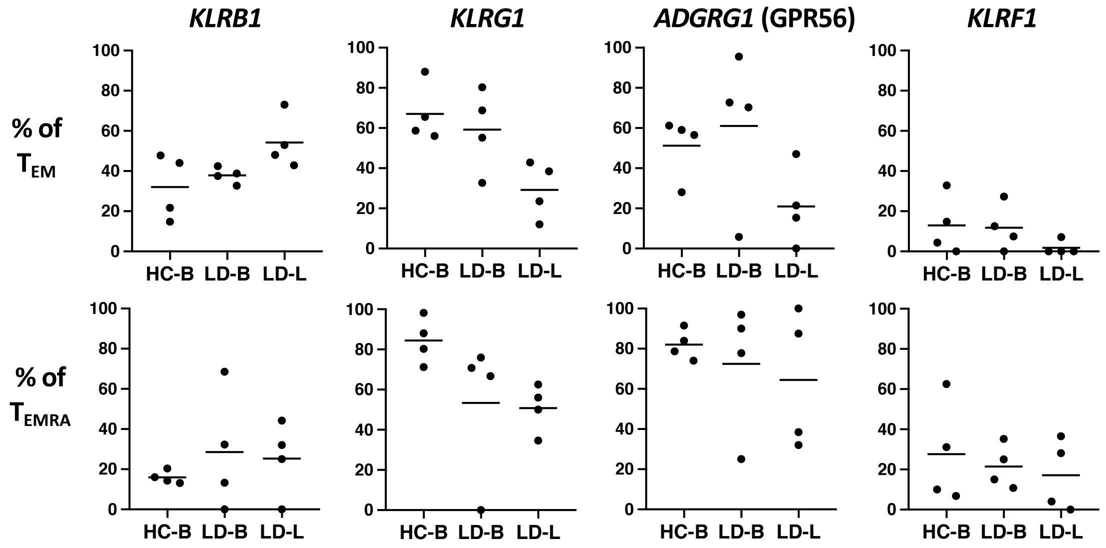

Frequency of T_{EM} and T_{EMRA} cells (scatter plots with median) transcribing *KLRB1, KLRG1, ADGRG1* (GPR56) and *KLRF1* from blood of healthy individuals (HC-B, n = 4) as well as blood (LD-B, n = 4) and liver samples (LD-L, n = 4) of patients with inflammatory biliary and hepatic diseases. Gene expression was determined by single cell qRT-PCR. Data are shown with mean.

Supplementry figure 5: Pre-gating strategy for flow cytometry investigations

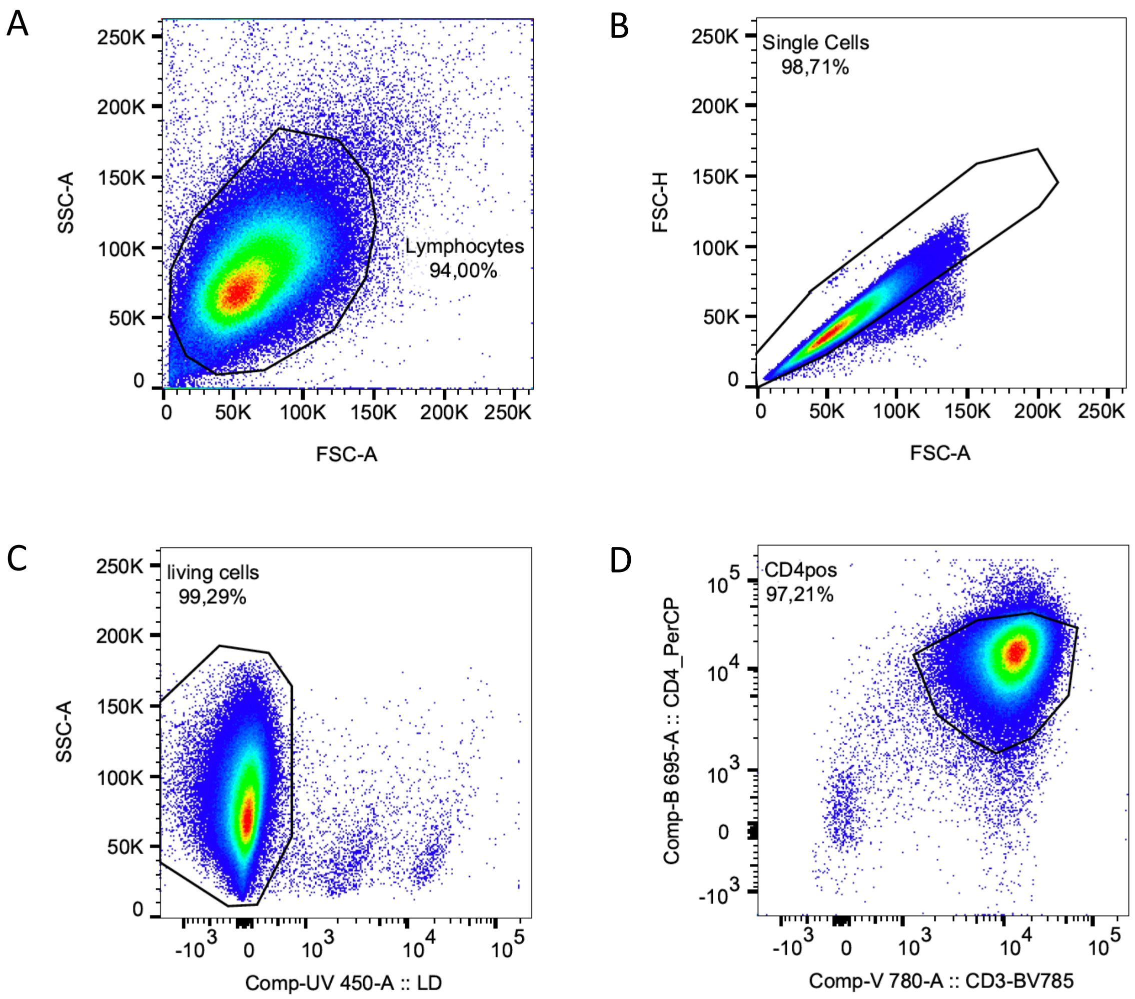

Exemplary hierarchical gating strategy to identify CD4+ T cells.

All samples were pre-gated on (A) lymphocytes followed by (B) FSC-H/FSC-A-based doublet exclusion. (C) Living cells were discriminated from dead cells by viability staining. (D) T cells within living cells were identified by expression of CD3 and CD4. Original dot plots for the identification of subsequent populations are shown in the specific figures.

Supplementary table 1: List of genes and their log2 fold expression up-regulation identified by intersection analysis (e.g. T_{EMRA}/T_{EM} vs. T_N/T_{CM}) of RNA microarray results from sorted human peripheral blood CD4+ T_N, T_{CM}, T_{EM} and T_{EMRA} cells. Rows filled in grey mark genes with specifically increased expression in T_{EM} cells compared to T_N cells.

	**Fold change**			
**Gene symbol**	T_{EMRA}/T_{EM} vs T_N/T_{CM} [log2]	T_{EM} vs T_N [log2]	T_{EMRA} vs T_N [log2]	**Gene name**
S1PR5	6.306	4.003	7.107	Sphingosine-1-Phosphate Receptor 5
GPR56	6.74	4.157	7.852	G protein-coupled receptor 56
CXCR1	6.263	3.176	7.814	C-X-C motif chemokine receptor 1
NKG7	6.295	4.589	7.779	natural killer cell granule protein 7
CMKLR1	5.682	3.709	6.92	Chemokine like receptor 1
SLAMF7	5.059	3.989	6.952	SLAM Family Member 7
FASLG	4.719	4.307	7.173	Fas ligand
KLRF1	3.805	1.689	4.646	killer cell lectin like receptor F1
OR10H2	3.855	2.211	4.739	olfactory receptor family 10 subfamily H member 2
ENPP5	3.58	1.884	4.826	ectonucleotide pyrophosphatase/phosphodiesterase 5
PCDHGA7	3.085	1.873	3.906	protocadherin gamma subfamily A, 7
CD300A	2.728	1.777	4.105	Cluster of Differentiation 300A
HRH3	2.325	1.442	2.994	Histamine H3 receptor
SLC4A4	2.585	1.235	3.412	Solute carrier family 4
FZD2	2.281	0.901	3.56	frizzled class receptor 2
HLA-DRB4	2.268	2.114	3.804	Major histocompatibility complex, class II, DR beta 4
PTCH1	2.295	0.842	2.773	patched 1
CD300C	2.404	1.59	3.795	Cluster of Differentiation 300C
KLRG1	2.515	2.618	3.924	killer cell lectin like receptor G1
LAG3	2.433	2.362	3.94	Lymphocyte-activation protein 3
HLA-DRB5	2.282	2.554	3.738	major histocompatibility complex, class II, DR beta 5
HLA-DRB1	2.137	2.156	3.197	major histocompatibility complex, class II, DR beta 1
SLC27A3	2.066	1.68	2.834	solute carrier family 27 member 3
MCTP2	1.708	0.828	2.399	multiple C2 and transmembrane domain containing 2
HLA-DRB5	2.025	2.25	3.532	major histocompatibility complex, class II, DR beta 5
FCRL3	1.466	2.173	3.557	Fc receptor like 3
HCST	1.505	0.888	2.034	hematopoietic cell signal transducer
LPCAT1	1.557	0.768	1.955	lysophosphatidylcholine acyltransferase 1
SPN	1.517	0.905	2.107	sialophorin
TLR3	1.682	2.621	3.778	toll like receptor 3
SIGLEC9	1.265	0.564	1.899	sialic acid binding Ig like lectin 9
SLC8A1	1.044	0.835	2.436	solute carrier family 8 member A1
CD99	1.509	1.435	2.598	Cluster of differentiation 99
LILRB3	1.177	0.7	1.912	leukocyte immunoglobulin like receptor B3
FCGR2A	1.041	0.349	2.059	Fc fragment of IgG receptor IIa
ITGAX	0.677	-0.013	1.335	integrin subunit alpha X
ERBB2	0.543	-0.783	1.032	erb-b2 receptor tyrosine kinase 2
TLR4	1.049	0.411	1.43	toll like receptor 4
SLC2A6	0.788	-0.12	1.108	solute carrier family 2 (facilitated glucose transporter), member 6
KIT	0.876	1.791	0.059	KIT proto-oncogene receptor tyrosine kinase
ASB2	0.619	1.78	1.39	ankyrin Repeat And SOCS Box Containing 2
PPARG	0.617	0.992	0.082	peroxisome proliferator activated receptor gamma
HLF	0.4	1.214	0.668	hepatic leukemia factor
NMU	0.661	1.997	1.026	Neuromedin U
KLRB1	0.287	0.704	0.518	killer cell lectin like receptor B1

Supplementary table 2: List of TaqMan Gene Expression Assays used within single cell qRT-PCR

No.	Gene Symbol	Transcript (Refseq)	Assay ID	Exon Boundary	Assay Location (bp)	Amplicon Length (bp)	Gene Length (bp)
1	AQP9	NM_020980	Hs01035888_m1	5-6	1065	98	3033
2	RETN	NM_020415	Hs00220767_m1	3-4	242	130	478
3	RNASE2	NM_002934	Hs00795553_s1	2-2	379	91	735
4	RNASE3	NM_002935	Hs03046240_s1	2-2	479	140	712
5	LTF	NM_002343	Hs00914330_m1	16-17	2340	92	2627
6	CA1	NM_001738	Hs01100176_m1	8-9	836	86	1278
7	LCN2	NM_005564	Hs01008571_m1	5-6	642	61	840
8	S100A9	NM_002965	Hs00610058_m1	2-3	188	83	586
9	C19orf59	NM_174918	Hs00545332_m1	1-2	121	109	1326
10	FPR1	NM_002029	Hs00181830_m1	1-2	88	132	1334
11	S100A8	NM_002964	Hs00374263_m1	1-2	148	70	532
12	FCGR1B	NM_001017986	Hs02341825_m1	4-5	615	86	1917
13	S100A12	NM_005621	Hs00194525_m1	1-2	46	63	466
14	MS4A4A	NM_024021	Hs00254770_m1	3-4	480	80	1814
15	GPER	NM_001039966	Hs01116133_m1	2-3	563	60	2981
16	APOBEC3H	NM_181773	Hs03989381_m1	4-5	670	107	1070
17	S1PR5	NM_030760	Hs00928195_s1	2-2	1977	86	2351
18	GPR56	NM_201525	Hs00938469_m1	13 - 14	2266	68	4133
19	NKG7	NM_005601	Hs01120688_g1	3-4	616	65	826
20	ADRB2	NM_000024	Hs00240532_s1	1-1	778	65	2058
21	CMKLR1	NM_004072	Hs03063327_m1	1-2	287	54	5420
22	PTGDR	NM_000953	Hs00235003_m1	1-2	950	72	2966
23	NMUR1	NM_006056	Hs00173804_m1	2-3	1014	60	3274
24	GPR114	NM_153837	Hs00542389_m1	10-11	1728	74	3848
25	LAIR2	NM_002288	Hs00357971_m1	4-5	536	71	702
26	CADM1	NM_014333	Hs00942509_m1	3-4	549	77	4324
27	KLRF1	NM_016523	Hs00212979_m1	3-4	398	70	1242
28	CD300A	NM_007261	Hs00381974_m1	3-4	856	88	1893
29	KLRG1	NM_005810	Hs00929964_m1	3-4	370	83	1815
30	CD300C	NM_006678	Hs01088618_m1	1-2	451	100	1602
31	TLR3	NM_003265	Hs01551078_m1	3-4	738	132	3057
32	KIT	NM_000222	Hs00922198_m1	16 - 17	2442	75	5190
33	ASB2	NM_016150	Hs00387867_m1	4-5	1225	55	2756
34	KLRB1	NM_002258	Hs00935894_m1	2-3	249	90	740
35	CDO1	NM_001801	Hs00156447_m1	1-2	482	66	1627
36	GPR44	NM_004778	Hs00173717_m1	1-2	105	71	2910
37	KRT1	NM_006121	Hs00196158_m1	6-7	1316	86	2451
38	*CA6*	*NM_001215*	Hs00154196_m1	2-3	316	77	1378
39	*SELL*	*NM_000655*	Hs01053460_m1	7-8	1288	79	2206
49	PTPRC(CD45RA?)	NM_001267798	Hs04189704_m1	2-3	280	57	1477
40	spike 1		AI0IX5E				
41	HPRT1		Hs02800695_m1	2-3	297	82	1435
42	B2M		Hs00984230_m1	3-4	431	81	987
43	FOXP3		Hs00203958_m1	9-10	1132	64	2292
44	EOMES	NM_005442	Hs00172872_m1	2-3	1458	81	3617
45	GATA3	NM_001002295	Hs00922328_m1	4-5	1485	86	3070

Supplementary table 3: Frequency of transcript expressing T cell populations upon single cell gene expression profiling

	Populations									
Gene	T_N		T_{CM}		T_{EM}		T_{EMRA}		T_{reg}	
	mean	SEM	mean	SEM	mean	SEM	mean	SEM	mean	SEM
RNASE2	27.34	6.38	8.33	2.63	16.60	7.00	11.36	3.70	18.23	5.66
RNASE3	22.44	6.74	7.15	2.15	19.64	6.56	15.41	3.94	19.27	5.60
LTF	0.00	0.00	0.00	0.00	0.00	0.00	0.00	0.00	10.16	5.08
S1PR5	1.67	0.83	0.00	0.00	2.86	0.84	6.46	1.24	0.00	0.00
NKG7	3.45	1.00	2.53	1.02	66.70	6.07	92.51	1.62	1.82	0.54
ADRB2	5.16	1.63	2.89	0.96	24.48	6.64	35.99	3.81	6.77	1.79
CMKLR1	3.45	1.00	0.00	0.00	20.38	1.54	30.32	4.15	1.65	0.31
PTGDR	5.24	1.70	8.43	0.60	23.65	0.42	28.32	1.93	0.39	0.20
NMUR1	0.00	0.00	0.36	0.18	4.77	0.46	5.18	1.50	0.39	0.20
GPR114	3.35	0.74	1.43	0.71	24.85	1.89	43.46	3.18	2.73	1.12
LAIR2	4.25	1.70	5.49	1.38	16.08	2.41	15.31	1.09	12.66	2.05
CADM1	0.00	0.00	3.71	0.72	9.74	2.19	15.47	2.32	3.99	0.91
KLRF1	1.67	0.83	0.00	0.00	12.98	3.65	27.61	6.41	0.39	0.20
CD300A	6.03	1.56	30.60	4.20	57.69	6.53	80.04	3.20	3.47	1.03
KLRG1	10.62	1.29	16.96	0.95	67.08	3.64	84.43	2.87	2.34	1.17
TLR3	0.00	0.00	2.90	0.60	6.57	1.12	5.60	0.68	0.00	0.00
ASB2	0.00	0.00	4.35	1.23	3.64	0.81	1.73	0.36	0.78	0.39
KLRB1	0.89	0.45	36.85	4.34	32.06	4.07	15.93	0.79	12.27	1.06
GPR44	0.00	0.00	3.96	1.73	1.09	0.54	0.00	0.00	1.56	0.78
KRT1	4.23	1.58	2.50	1.03	0.00	0.00	0.00	0.00	1.82	0.54
SELL	93.10	2.00	63.29	1.13	18.49	4.12	36.00	6.75	81.75	2.55
CD45RA	89.72	4.29	12.09	4.05	11.91	3.16	75.29	4.43	18.96	5.03
HPRT	23.05	1.98	30.70	2.58	26.76	2.13	26.07	2.09	38.86	3.04
ß2M	100.00	0.00	100.00	0.00	100.00	0.00	100.00	0.00	100.00	0.00
FOP3	0.68	0.34	4.44	1.27	2.66	0.51	0.50	0.25	87.03	2.64
EOMES	5.02	0.55	2.92	0.58	42.99	2.77	55.04	3.32	1.17	0.59
GATA3	40.11	2.90	70.91	0.83	63.30	2.78	59.45	3.49	66.97	3.13
RORC	0.00	0.00	6.89	1.51	6.20	1.05	1.27	0.40	4.60	1.00
CX3CR1	3.45	1.00	1.43	0.71	59.46	4.77	73.76	3.66	2.69	0.67
APOBEC3H	0.00	0.00	6.10	1.94	13.23	0.56	12.66	2.21	2.82	0.65
KIT	0.00	0.00	0.00	0.00	1.00	0.50	0.00	0.00	0.00	0.00
GPR56	4.35	0.83	2.14	1.07	51.18	3.89	82.04	1.88	0.39	0.20

Supplementary table 4:

Overview on reported expression profiles of known checkpoint molecules & killer-like receptors as well as GPR56 in human and murine T cells. The values *low, medium* and *high* indicate the relative distribution of a given marker within $CD4^+$ and $CD8^+$ T cell subsets regardless of the absolute frequency of positive cells. *NDA* = no data available

**marker**		**activated**		**memory**								**dysfunctional / exhausted**		**References**
		**human**	**murine**	**human**				**murine**				**human**	**murine**	
				**overall**	T_{CM}	T_{EM}	T_{EMRA}	**overall**	T_{CM}	T_{EM}	T_{EMRA}			
**PD1**	*CD4*	medium	high	high	low/medium	medium/high	medium/high	medium	medium/low	high	*NDA*	variable	high	[1-11]
	CD8	*NDA*	*NDA*	*NDA*	medium	medium/high	high	*NDA*	*NDA*	*NDA*	*NDA*	high/variable	high	[11-15]
**TIGIT**	*CD4*	low/medium	medium	medium/high	medium/high	medium/low	low	high	*NDA*	*NDA*	*NDA*	high	high	[1, 6, 8, 11, 16-20]
	CD8	high	high	medium	low	high	medium	medium	low	high	*NDA*	high/variable	high	[11, 14, 21, 22]
**TIM3**	*CD4*	medium	medium/high	medium	low	high	medium	*NDA*	*NDA*	*NDA*	*NDA*	high	high	[6, 7, 9, 11, 23-27]
	CD8	medium	medium	low	low	low	*NDA*	medium	low/medium	high	*NDA*	high	high	[11, 15, 28-30]
**LAG3**	*CD4*	low/medium	high	medium	medium	medium/high	medium	low/medium	low	medium	*NDA*	high	high	[3, 6, 8-11, 31-33]
	CD8	medium/high	medium	medium	low/medium	medium	high	low	*NDA*	*NDA*	*NDA*	high	high	[6, 10, 11, 13, 15, 28]
**2B4**	*CD4*	low	low	low	low	low	*NDA*	low	*NDA*	*NDA*	*NDA*	low/medium	medium/high	[8, 9, 34-36]
	CD8	*NDA*	high	medium	low	medium	high	*NDA*	*NDA*	high	*NDA*	*NDA*	high	[12, 13, 15, 37-39]
**CD160**	*CD4*	low	*NDA*	low	*NDA*	high	*NDA*	*NDA*	*NDA*	*NDA*	*NDA*	low	*NDA*	[8, 40-44]
	CD8	high	high	*NDA*	low	low/medium	medium/high	*NDA*	medium	medium	*NDA*	medium/high	*NDA*	[12, 13, 15, 39]
**KLRB1**	*CD4*	*NDA*	*NDA*	high	medium	high	*NDA*	*NDA*	*NDA*	*NDA*	*NDA*	low	*NDA*	[45-48]
	CD8	low	*NDA*	*NDA*	medium	high	high	*NDA*	*NDA*	*NDA*	*NDA*	*NDA*	*NDA*	[49]
**KLRG1**	*CD4*	*NDA*	low	*NDA*	low	medium/high	high	*NDA*	*NDA*	*NDA*	high	low	low	[50-56]
	CD8	low	medium/high	high	low/medium	medium/high	high	*NDA*	low	medium	medium	*NDA*	variable	[12, 13, 15, 57-60]
**GPR56**	*CD4*	*NDA*	*NDA*	*NDA*	low	variable	high	*NDA*	*NDA*	*NDA*	*NDA*	*NDA*	*NDA*	[5]
	CD8	*NDA*	*NDA*	*NDA*	*NDA*	*NDA*	*NDA*	*NDA*	*NDA*	*NDA*	*NDA*	medium	*NDA*	[61]
**KLRF1**	*CD4*	*NDA*	*NDA*	*NDA*	*NDA*	*NDA*	*NDA*	*NDA*	*NDA*	*NDA*	*NDA*	*NDA*	*NDA*	
	CD8	high	*NDA*	*NDA*	*NDA*	*NDA*	*NDA*	*NDA*	*NDA*	*NDA*	*NDA*	*NDA*	*NDA*	[62]

References:

1. Josefsson, S.E., et al., *TIGIT and PD-1 mark intratumoral T cells with reduced effector function in B-cell non-Hodgkin lymphoma.* Cancer Immunol Res, 2019. **18**: p. 2326-6066.
2. Cockerham, L.R., et al., *Programmed death-1 expression on CD4(+) and CD8(+) T cells in treated and untreated HIV disease.* AIDS., 2014. **28**(12): p. 1749-58. doi: 10.1097/QAD.0000000000000314.
3. Crawford, A., et al., *Molecular and transcriptional basis of CD4(+) T cell dysfunction during chronic infection.* Immunity., 2014. **40**(2): p. 289-302. doi: 10.1016/j.immuni.2014.01.005. Epub 2014 Feb 13.
4. Rosignoli, G., et al., *Programmed death (PD)-1 molecule and its ligand PD-L1 distribution among memory CD4 and CD8 T cell subsets in human immunodeficiency virus-1-infected individuals.* Clin Exp Immunol., 2009. **157**(1): p. 90-7. doi: 10.1111/j.1365-2249.2009.03960.x.
5. Agata, Y., et al., *Expression of the PD-1 antigen on the surface of stimulated mouse T and B lymphocytes.* Int Immunol., 1996. **8**(5): p. 765-72.
6. Anderson, A.C., N. Joller, and V.K. Kuchroo, *Lag-3, Tim-3, and TIGIT: Co-inhibitory Receptors with Specialized Functions in Immune Regulation.* Immunity., 2016. **44**(5): p. 989-1004. doi: 10.1016/j.immuni.2016.05.001.
7. McMahan, R.H., et al., *Tim-3 expression on PD-1+ HCV-specific human CTLs is associated with viral persistence, and its blockade restores hepatocyte-directed in vitro cytotoxicity.* J Clin Invest., 2010. **120**(12): p. 4546-57. doi: 10.1172/JCI43127. Epub 2010 Nov 15.
8. Fromentin, R., et al., *CD4+ T Cells Expressing PD-1, TIGIT and LAG-3 Contribute to HIV Persistence during ART.* PLoS Pathog., 2016. **12**(7): p. e1005761. doi: 10.1371/journal.ppat.1005761. eCollection 2016 Jul.
9. Goding, S.R., et al., *Restoring immune function of tumor-specific CD4+ T cells during recurrence of melanoma.* J Immunol., 2013. **190**(9): p. 4899-909. doi: 10.4049/jimmunol.1300271. Epub 2013 Mar 27.
10. Tian, X., et al., *The upregulation of LAG-3 on T cells defines a subpopulation with functional exhaustion and correlates with disease progression in HIV-infected subjects.* J Immunol., 2015. **194**(8): p. 3873-82. doi: 10.4049/jimmunol.1402176. Epub 2015 Mar 16.
11. Tassi, E., et al., *Early Effector T Lymphocytes Coexpress Multiple Inhibitory Receptors in Primary Non-Small Cell Lung Cancer.* Cancer Res., 2017. **77**(4): p. 851-861. doi: 10.1158/0008-5472.CAN-16-1387. Epub 2016 Dec 15.
12. Bengsch, B., et al., *Coexpression of PD-1, 2B4, CD160 and KLRG1 on exhausted HCV-specific CD8+ T cells is linked to antigen recognition and T cell differentiation.* PLoS Pathog., 2010. **6**(6): p. e1000947. doi: 10.1371/journal.ppat.1000947.
13. Blackburn, S.D., et al., *Coregulation of CD8+ T cell exhaustion by multiple inhibitory receptors during chronic viral infection.* Nat Immunol., 2009. **10**(1): p. 29-37. doi: 10.1038/ni.1679. Epub 2008 Nov 30.
14. Chauvin, J.M., et al., *TIGIT and PD-1 impair tumor antigen-specific CD8(+) T cells in melanoma patients.* J Clin Invest., 2015. **125**(5): p. 2046-58. doi: 10.1172/JCI80445. Epub 2015 Apr 13.
15. Legat, A., et al., *Inhibitory Receptor Expression Depends More Dominantly on Differentiation and Activation than "Exhaustion" of Human CD8 T Cells.* Front Immunol., 2013. **4:455.**(doi): p. 10.3389/fimmu.2013.00455. eCollection 2013.
16. Catakovic, K., et al., *TIGIT expressing CD4+T cells represent a tumor-supportive T cell subset in chronic lymphocytic leukemia.* Oncoimmunology., 2017. **7**(1): p. e1371399. doi: 10.1080/2162402X.2017.1371399. eCollection 2017.
17. Yu, X., et al., *The surface protein TIGIT suppresses T cell activation by promoting the generation of mature immunoregulatory dendritic cells.* Nat Immunol., 2009. **10**(1): p. 48-57. doi: 10.1038/ni.1674. Epub 2008 Nov 16.
18. Boles, K.S., et al., *A novel molecular interaction for the adhesion of follicular CD4 T cells to follicular DC.* Eur J Immunol., 2009. **39**(3): p. 695-703. doi: 10.1002/eji.200839116.

19. Joller, N., et al., *Cutting edge: TIGIT has T cell-intrinsic inhibitory functions.* J Immunol., 2011. **186**(3): p. 1338-42. doi: 10.4049/jimmunol.1003081. Epub 2011 Jan 3.
20. Levin, S.D., et al., *Vstm3 is a member of the CD28 family and an important modulator of T-cell function.* Eur J Immunol., 2011. **41**(4): p. 902-15. doi: 10.1002/eji.201041136. Epub 2011 Mar 18.
21. Johnston, R.J., et al., *The immunoreceptor TIGIT regulates antitumor and antiviral CD8(+) T cell effector function.* Cancer Cell., 2014. **26**(6): p. 923-937. doi: 10.1016/j.ccell.2014.10.018. Epub 2014 Nov 26.
22. Song, Y., et al., *T-cell Immunoglobulin and ITIM Domain Contributes to CD8(+) T-cell Immunosenescence.* Aging Cell., 2018. **17(2).**(doi): p. 10.1111/acel.12716. Epub 2018 Jan 19.
23. Hastings, W.D., et al., *TIM-3 is expressed on activated human CD4+ T cells and regulates Th1 and Th17 cytokines.* Eur J Immunol., 2009. **39**(9): p. 2492-501. doi: 10.1002/eji.200939274.
24. Jones, R.B., et al., *Tim-3 expression defines a novel population of dysfunctional T cells with highly elevated frequencies in progressive HIV-1 infection.* J Exp Med., 2008. **205**(12): p. 2763-79. doi: 10.1084/jem.20081398. Epub 2008 Nov 10.
25. Qiu, Y., et al., *Tim-3-expressing CD4+ and CD8+ T cells in human tuberculosis (TB) exhibit polarized effector memory phenotypes and stronger anti-TB effector functions.* PLoS Pathog, 2012. **8**(11): p. e1002984. doi: 10.1371/journal.ppat.1002984. Epub 2012 Nov 8.
26. Kim, J.E., et al., *Combination Therapy with Anti-PD-1, Anti-TIM-3, and Focal Radiation Results in Regression of Murine Gliomas.* Clin Cancer Res., 2017. **23**(1): p. 124-136. doi: 10.1158/1078-0432.CCR-15-1535. Epub 2016 Jun 29.
27. Zhu, C., et al., *An IL-27/NFIL3 signalling axis drives Tim-3 and IL-10 expression and T-cell dysfunction.* Nat Commun., 2015. **6:6072.**(doi): p. 10.1038/ncomms7072.
28. Kim, J., et al., *Memory programming in CD8(+) T-cell differentiation is intrinsic and is not determined by CD4 help.* Nat Commun., 2015. **6:7994.**(doi): p. 10.1038/ncomms8994.
29. Sakuishi, K., et al., *Targeting Tim-3 and PD-1 pathways to reverse T cell exhaustion and restore anti-tumor immunity.* J Exp Med., 2010. **207**(10): p. 2187-94. doi: 10.1084/jem.20100643. Epub 2010 Sep 6.
30. Zhou, Q., et al., *Coexpression of Tim-3 and PD-1 identifies a CD8+ T-cell exhaustion phenotype in mice with disseminated acute myelogenous leukemia.* Blood., 2011. **117**(17): p. 4501-10. doi: 10.1182/blood-2010-10-310425. Epub 2011 Mar 8.
31. Workman, C.J., et al., *Phenotypic analysis of the murine CD4-related glycoprotein, CD223 (LAG-3).* Eur J Immunol., 2002. **32**(8): p. 2255-63. doi: 10.1002/1521-4141(200208)32:8<2255::AID-IMMU2255>3.0.CO;2-A.
32. Villegas-Mendez, A., et al., *Long-Lived CD4+IFN-gamma+ T Cells rather than Short-Lived CD4+IFN-gamma+IL-10+ T Cells Initiate Rapid IL-10 Production To Suppress Anamnestic T Cell Responses during Secondary Malaria Infection.* J Immunol., 2016. **197**(8): p. 3152-3164. doi: 10.4049/jimmunol.1600968. Epub 2016 Sep 14.
33. Triebel, F., et al., *LAG-3, a novel lymphocyte activation gene closely related to CD4.* J Exp Med., 1990. **171**(5): p. 1393-405.
34. Nakajima, H., et al., *Activating interactions in human NK cell recognition: the role of 2B4-CD48.* Eur J Immunol., 1999. **29**(5): p. 1676-83. doi: 10.1002/(SICI)1521-4141(199905)29:05<1676::AID-IMMU1676>3.0.CO;2-Y.
35. Noval Rivas, M., et al., *NK cell regulation of CD4 T cell-mediated graft-versus-host disease.* J Immunol., 2010. **184**(12): p. 6790-8. doi: 10.4049/jimmunol.0902598. Epub 2010 May 19.
36. Romero, X., et al., *Differential expression of SAP and EAT-2-binding leukocyte cell-surface molecules CD84, CD150 (SLAM), CD229 (Ly9) and CD244 (2B4).* Tissue Antigens., 2004. **64**(2): p. 132-44. doi: 10.1111/j.1399-0039.2004.00247.x.
37. Kambayashi, T., et al., *Cutting edge: Regulation of CD8(+) T cell proliferation by 2B4/CD48 interactions.* J Immunol., 2001. **167**(12): p. 6706-10.
38. Speiser, D.E., et al., *The activatory receptor 2B4 is expressed in vivo by human CD8+ effector alpha beta T cells.* J Immunol., 2001. **167**(11): p. 6165-70.

39. Tsujimura, K., et al., *Characterization of murine CD160+ CD8+ T lymphocytes.* Immunol Lett., 2006. **106**(1): p. 48-56. doi: 10.1016/j.imlet.2006.04.006. Epub 2006 May 11.
40. El-Far, M., et al., *CD160 isoforms and regulation of CD4 and CD8 T-cell responses.* J Transl Med., 2014. **12:217.**(doi): p. 10.1186/s12967-014-0217-y.
41. Paris, R.M., et al., *Selective Loss of Early Differentiated, Highly Functional PD1high CD4 T Cells with HIV Progression.* PLoS One., 2015. **10**(12): p. e0144767. doi: 10.1371/journal.pone.0144767. eCollection 2015.
42. Porichis, F., et al., *Responsiveness of HIV-specific CD4 T cells to PD-1 blockade.* Blood., 2011. **118**(4): p. 965-74. doi: 10.1182/blood-2010-12-328070. Epub 2011 Jun 7.
43. Xia, H., et al., *Elevated Level of CD4(+) T Cell Immune Activation in Acutely HIV-1-Infected Stage Associates With Increased IL-2 Production and Cycling Expression, and Subsequent CD4(+) T Cell Preservation.* Front Immunol., 2018. **9:616.**(doi): p. 10.3389/fimmu.2018.00616. eCollection 2018.
44. Sako, N., et al., *Membrane expression of NK receptors CD160 and CD158k contributes to delineate a unique CD4+ T-lymphocyte subset in normal and mycosis fungoides skin.* Cytometry A., 2014. **85**(10): p. 869-82. doi: 10.1002/cyto.a.22512. Epub 2014 Jul 18.
45. Hassouneh, F., et al., *Differential Effect of Cytomegalovirus Infection with Age on the Expression of CD57, CD300a, and CD161 on T-Cell Subpopulations.* Front Immunol., 2017. **8:649.**(doi): p. 10.3389/fimmu.2017.00649. eCollection 2017.
46. Iliopoulou, E.G., et al., *Increased frequency of CD4+ cells expressing CD161 in cancer patients.* Clin Cancer Res., 2006. **12**(23): p. 6901-9. doi: 10.1158/1078-0432.CCR-06-0977.
47. Lanier, L.L., C. Chang, and J.H. Phillips, *Human NKR-P1A. A disulfide-linked homodimer of the C-type lectin superfamily expressed by a subset of NK and T lymphocytes.* J Immunol., 1994. **153**(6): p. 2417-28.
48. Rodriguez, M.W., et al., *Differential gene expression by integrin beta 7+ and beta 7- memory T helper cells.* BMC Immunol., 2004. **5:13.**(doi): p. 10.1186/1471-2172-5-13.
49. Northfield, J.W., et al., *CD161 expression on hepatitis C virus-specific CD8+ T cells suggests a distinct pathway of T cell differentiation.* Hepatology., 2008. **47**(2): p. 396-406. doi: 10.1002/hep.22040.
50. Beyersdorf, N., et al., *Characterization of mouse CD4 T cell subsets defined by expression of KLRG1.* Eur J Immunol., 2007. **37**(12): p. 3445-54. doi: 10.1002/eji.200737126.
51. Cyktor, J.C., et al., *Killer cell lectin-like receptor G1 deficiency significantly enhances survival after Mycobacterium tuberculosis infection.* Infect Immun., 2013. **81**(4): p. 1090-9. doi: 10.1128/IAI.01199-12. Epub 2013 Jan 22.
52. Di Mitri, D., et al., *Reversible senescence in human CD4+CD45RA+CD27- memory T cells.* J Immunol., 2011. **187**(5): p. 2093-100. doi: 10.4049/jimmunol.1100978. Epub 2011 Jul 25.
53. Lindenstrom, T., et al., *Control of chronic mycobacterium tuberculosis infection by CD4 KLRG1- IL-2-secreting central memory cells.* J Immunol., 2013. **190**(12): p. 6311-9. doi: 10.4049/jimmunol.1300248. Epub 2013 May 15.
54. Raziorrouh, B., et al., *Inhibitory phenotype of HBV-specific CD4+ T-cells is characterized by high PD-1 expression but absent coregulation of multiple inhibitory molecules.* PLoS One., 2014. **9**(8): p. e105703. doi: 10.1371/journal.pone.0105703. eCollection 2014.
55. Reiley, W.W., et al., *Distinct functions of antigen-specific CD4 T cells during murine Mycobacterium tuberculosis infection.* Proc Natl Acad Sci U S A., 2010. **107**(45): p. 19408-13. doi: 10.1073/pnas.1006298107. Epub 2010 Oct 20.
56. Voehringer, D., M. Koschella, and H. Pircher, *Lack of proliferative capacity of human effector and memory T cells expressing killer cell lectinlike receptor G1 (KLRG1).* Blood., 2002. **100**(10): p. 3698-702. doi: 10.1182/blood-2002-02-0657. Epub 2002 Jul 12.
57. Joshi, N.S., et al., *Inflammation directs memory precursor and short-lived effector CD8(+) T cell fates via the graded expression of T-bet transcription factor.* Immunity., 2007. **27**(2): p. 281-95. doi: 10.1016/j.immuni.2007.07.010.

58. Angelosanto, J.M., et al., *Progressive loss of memory T cell potential and commitment to exhaustion during chronic viral infection.* J Virol., 2012. **86**(15): p. 8161-70. doi: 10.1128/JVI.00889-12. Epub 2012 May 23.
59. Sarkar, S., et al., *Functional and genomic profiling of effector CD8 T cell subsets with distinct memory fates.* J Exp Med., 2008. **205**(3): p. 625-40. doi: 10.1084/jem.20071641. Epub 2008 Mar 3.
60. Voehringer, D., et al., *Viral infections induce abundant numbers of senescent CD8 T cells.* J Immunol., 2001. **167**(9): p. 4838-43.
61. Peng, Y.M., et al., *Specific expression of GPR56 by human cytotoxic lymphocytes.* J Leukoc Biol., 2011. **90**(4): p. 735-40. doi: 10.1189/jlb.0211092. Epub 2011 Jul 1.
62. Kuttruff, S., et al., *NKp80 defines and stimulates a reactive subset of CD8 T cells.* Blood., 2009. **113**(2): p. 358-69. doi: 10.1182/blood-2008-03-145615. Epub 2008 Oct 15.
